# Cadmium Inhibits Proliferation of Human Bronchial Epithelial BEAS-2B Cells Through Inducing Ferroptosis via Targeted Regulation of the Nrf2/SLC7A11/GPX4 Pathway

**DOI:** 10.3390/ijms26157204

**Published:** 2025-07-25

**Authors:** Huan Li, Zixin Qiu, Long Chen, Tianbao Zhang, Diandian Wei, Xue Chen, Yun Wang

**Affiliations:** School of Public Health, Bengbu Medical University, Bengbu 233030, China; lh_1012022@163.com (H.L.); m15651958637@163.com (Z.Q.); 20241053029@stu.bbmu.edu.cn (L.C.); 18655793650@163.com (T.Z.); diandianwei@163.com (D.W.); 2014033@bbmu.edu.cn (X.C.)

**Keywords:** cadmium, BEAS-2B cells, ferroptosis, Nrf2/SLC7A11/GPX4 pathway, luteolin

## Abstract

Cadmium (Cd)-induced pulmonary toxicity is closely associated with ferroptosis, a regulated form of cell death characterized by iron-dependent lipid peroxidation (LPO). Luteolin (Lut) is a natural flavonoid compound that exists in many plants. In this study, we used human bronchial epithelial BEAS-2B cells to explore the impact of ferroptosis in the inhibition of Cd-induced BEAS-2B cells proliferation. BEAS-2B cells were exposed to Cd (5 μM) with/without Lut (10 μM), ferroptosis modulators (Ferrostatin-1 (Fer-1)/Erastin), or nuclear factor erythroid 2-related factor 2 (Nrf2) regulators (tert-butylhydroquinone (TBHQ)/ML385). Viability, iron content, reactive oxygen species (ROS), LPO, mitochondrial membrane potential (MMP), and glutathione peroxidase (GSH-PX) activity were assessed. Exposure to Cd significantly decreased cell viability, increased intracellular iron levels, ROS production, and LPO activity, while simultaneously reducing MMP and GSH-PX activity. Fer-1 mitigated Cd-induced cytotoxicity, but Erastin intensified these effects. Mechanistically, Cd exposure suppressed the Nrf2/Solute Carrier Family 7 Member 11 (SLC7A11)/glutathione peroxidase 4 (GPX4) signaling pathway, which plays a crucial role in maintaining redox homeostasis. Activation of Nrf2 using TBHQ mitigated oxidative stress and upregulated the expression of key proteins within this pathway, while inhibition of Nrf2 with ML385 exacerbated cellular damage. Notably, Lut treatment could significantly alleviate Cd-induced cytotoxicity, oxidative stress, and downregulation of Nrf2/SLC7A11/GPX4 proteins. These findings demonstrate that ferroptosis is a critical mechanism underlying Cd-mediated lung epithelial injury and identify Lut as a promising therapeutic candidate via its activation of Nrf2-driven antioxidant defense mechanisms. This study provides novel insights into molecular targets for the prevention and treatment of Cd-associated pulmonary disorders.

## 1. Introduction

The accelerating pace of industrialization and urbanization has precipitated a global environmental challenge due to widespread heavy metal contamination. Among these toxic elements, cadmium (Cd) poses a serious health threat owing to its multifaceted exposure routes, including respiratory inhalation, dietary consumption, and dermal penetration [1]. Occupational hazards are particularly important because airborne Cd particulates preferentially deposit in pulmonary tissues, initiating progressive respiratory dysfunction [2]. Alarmingly, tobacco smoke constitutes a major non-occupational exposure source, with epidemiological data revealing daily Cd absorption of approximately 1 μg from smoking 20 cigarettes [3]. Extensive data has verified that the lungs are one of the primary organs for Cd accumulation, where accumulated Cd disrupts pulmonary tissue homeostasis, exacerbates respiratory disorders, and elevates the risk of lung cancer [4]. These findings collectively underscore the pulmonary toxicity of Cd exposure.

Recent research underscores the critical significance of oxidative stress in Cd-induced pulmonary injury [5]. Oxidative stress, characterized by excessive reactive oxygen species (ROS), mitochondrial dysfunction, and disruption of calcium (Ca^2+^) homeostasis, disrupts the body’s redox balance [6]. Interestingly, ferroptosis, a unique kind of controlled cell death marked by dysregulation of iron metabolism and lipid peroxidation (LPO), can be brought on by an excess of ROS [7]. Emerging evidence demonstrates that exposure to environmental contaminants may trigger ferroptotic cell death pathways. For instance, arsenic can enhance ferritinophagy in chicken liver cells to induce ferroptosis [8], and mercuric chloride triggers hepatic ferroptosis by inducing LPO and iron overload, downregulating GPX4 and SLC7A11 expression and upregulating TRF1 expression [9]. Cd has also been shown to cause organs like the testes and liver to go through ferroptosis [10,11]. Therefore, we hypothesize that Cd-induced pulmonary damage may be closely associated with ferroptotic mechanisms.

Nuclear factor erythroid 2-related factor 2 (Nrf2), the principal transcriptional regulator of the cellular antioxidant response, sustains redox equilibrium by modulating antioxidant response elements and crucial to ferroptosis regulation [12]. Glutathione peroxidase 4 (GPX4) performs its antioxidant role via the glutathione-dependent enzymatic transformation of harmful lipid peroxidation derivatives into non-toxic substances, therefore blocking the LPO chain reaction and inhibiting ferroptosis [13]. Glutamine metabolism serves as a crucial regulatory nexus in the Nrf2/GPX4 pathway. Research indicates that the deprivation of glutamine catabolism can markedly diminish GPX4 levels, whereas the inhibition of glutamine metabolism can increase chemotherapy sensitivity by impairing Nrf2-mediated antioxidant defenses [14]. Recent studies indicate that Cd modulates glutamine metabolism via the endoplasmic reticulum stress/HMGA2 axis: it upregulates ASCT2 and ASNS to enhance cytoplasmic glutamine usage while concurrently inhibiting the expression of mitochondrial glutaminase GLS1 [15]. This metabolic reprogramming may hinder glutamine catabolism, thereby restricting the availability of reducing equivalents necessary for GPX4 activity. Nrf2 inactivation impairs the antioxidant defense system, diminishes the expression of SLC7A11 and GPX4, and elevates lipid peroxidation [16]. Research by Li et al. indicates that Cd exacerbates oxidative damage by decreasing antioxidant enzyme activity, augmenting ROS levels, and down-regulating Nrf2 proteins [17]. Consequently, we hypothesize that Cd-induced pulmonary illness may facilitate the accumulation of lipid peroxidation and ferroptosis in cells by obstructing the Nrf2/SLC7A11/GPX4 pathway.

Natural phytochemicals are gaining increased attention for their potential to mitigate Cd-induced pulmonary toxicity. Luteolin (Lut) is a natural flavonoid found in plants such as honeysuckle, chrysanthemum, and celery that possesses anti-inflammatory, antibacterial, and antioxidant properties [18]. Previous studies show that Lut can attenuate mercury-induced lung injury via Nrf2 activation and NF-κB inhibition, and it can protect against chromium-induced bronchial epithelial cell malignant transformation [19,20]. The preliminary findings validated Lut’s protective effect against Cd-induced damage in BEAS-2B cells [21], although its role in the control of ferroptosis remains inadequately comprehended. The present work constructed an in vitro model of Cd injury to explore whether exposure to Cd causes ferroptosis in bronchial epithelial BEAS-2B cells to evaluate the effect of Lut intervention and elucidate the underlying mechanisms.

## 2. Results

### 2.1. Cadmium (Cd) Inhibits BEAS-2B Cell Viability

**Figure 1:**
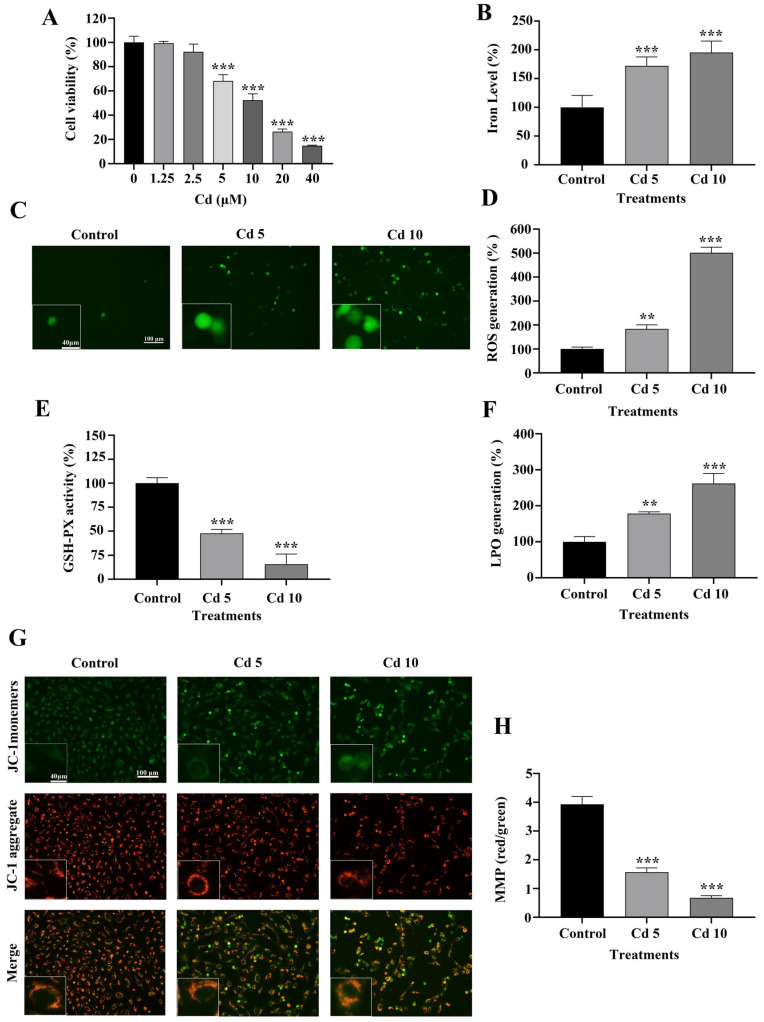
Cd Induces Ferroptosis in BEAS-2B Cells. (**A**) Cell viability was evaluated via MTT assay following treatment with different doses of Cd (0, 1.25, 2.5, 5, 10, 20, 40 μM) over 24 h. (**B**) Intracellular iron concentrations were quantified in BEAS-2B cells subjected to 5 μM and 10 μM Cd over 24 h. (**C**,**D**) ROS levels were detected using fluorescence microscopy. (**E**) GSH-PX activity and (**F**) LPO content were measured in cell lysates. (**G**,**H**) Mitochondrial membrane potential (MMP) was evaluated utilizing the JC-1 probe. The data is presented as the mean ± SD. Cd 5: 5 μM Cd, Cd 10: 10 μM Cd. ** *p* < 0.01, *** *p* < 0.001.

To delve into the suppressive influence of Cd on the proliferation of BEAS-2B cells, we assessed cell viability using the MTT assay, following treatment with various concentrations of CdCl_2_ (0, 1.25, 2.5, 5, 10, 20, and 40 μM). The results demonstrated that Cd significantly reduced BEAS-2B cell viability at concentrations of 5 μM and above, in a comparison with the control group (*p* < 0.001) (Figure 1A).

### 2.2. Cd Induces Ferroptosis by Regulating the Levels of LPO, GSH-PX and MMP in BEAS-2B Cells

An iron detection test evaluated intracellular iron levels, a ferroptosis marker, after Cd exposure. As shown in Figure 1B, Cd treatment significantly increased intracellular iron levels in BEAS-2B cells (*p* < 0.001). ROS accumulation is closely associated with ferroptosis induction. We observed that Cd exposure led to a substantial elevation of intracellular ROS levels, with statistical significance observed at higher Cd concentrations (*p* < 0.01) (Figure 1C,D). LPO and GSH-PX activity were further assessed to evaluate the extent of ferroptosis. Cd exposure resulted in significant increased LPO levels and markedly decreased GSH-PX activity (*p* < 0.01) (Figure 1E,F). Mitochondrial dysfunction is a key event in ferroptosis progression. In comparison to the control group, we observed a markedly lower MMP in BEAS-2B cells treated with Cd (*p* < 0.001) (Figure 1G,H). Collectively, these findings demonstrated that exposure to Cd causes ferroptosis in BEAS-2B cells by disrupting iron homeostasis, increasing ROS production, promoting LPO, and impairing mitochondrial function.

### 2.3. Fer-1 and Erastin Modulate Cd-Induced Ferroptosis in BEAS-2B Cells

**Figure 2:**
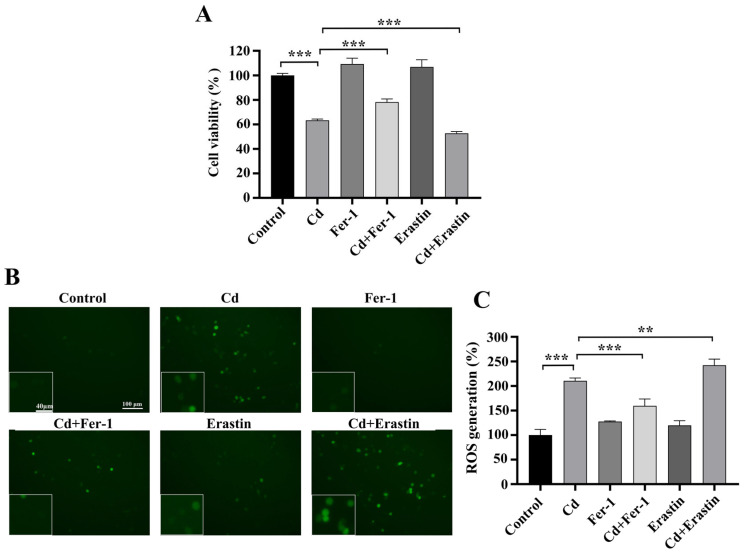
Effects of Fer-1 and Erastin on Cd-Induced BEAS-2B Cell Viability and ROS Generation. (**A**) Cell viability was assessed by the MTT test. (**B**,**C**) ROS generation was evaluated via fluorescence microscopy. The data is presented as the mean ± SD. Cd: 5 μM, Fer-1: 15 μM, Erastin: 1 μM. ** *p* < 0.01, *** *p* < 0.001.

**Figure 3:**
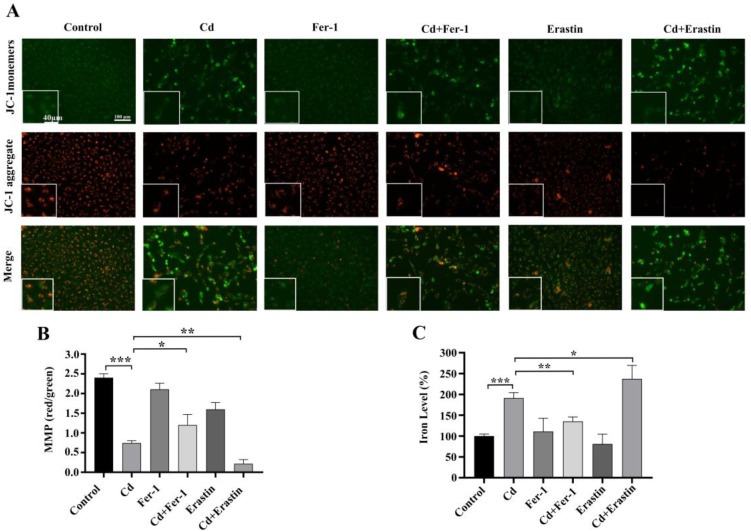
Impact of Fer-1 and Erastin on Cd-Induced Alterations in BEAS-2B Cell MMP and Iron Levels. (**A**,**B**) MMP was evaluated utilizing the JC-1 probe. (**C**) Intracellular iron levels were measured. The data is presented as the mean ± SD. Cd: 5 μM, Fer-1: 15 μM, Erastin: 1 μM. * *p* < 0.05, ** *p* < 0.01, *** *p* < 0.001.

**Figure 4:**
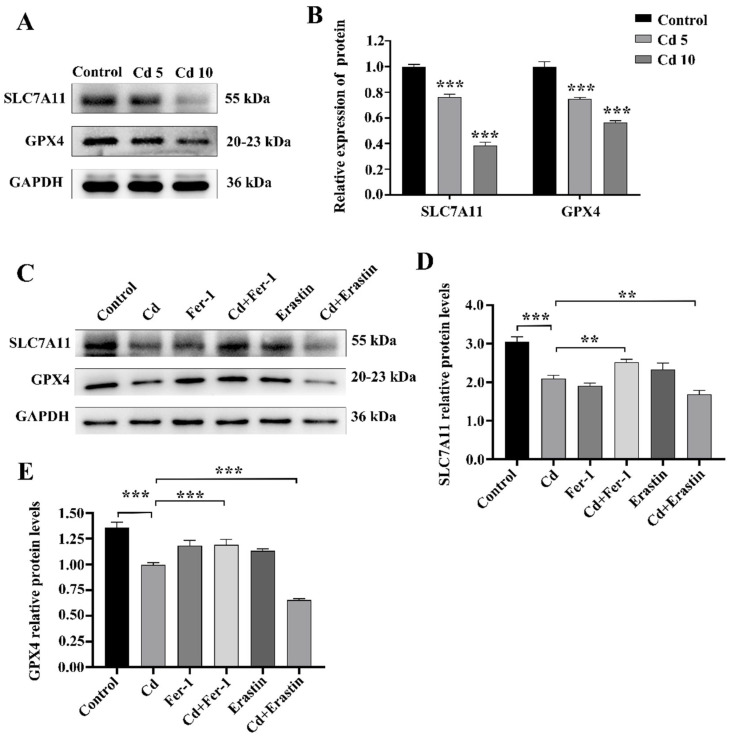
Impact of Fer1 and Erastin on Cd-Induced Ferroptosis-Associated Protein Expression in BEAS-2B Cells. (**A**,**B**) SLC7A11 and GPX4 protein expression Western blot analysis. (**C**–**E**) Protein expression of SLC7A11 and GPX4 following Fer-1 or Erastin treatment. The data is presented as the mean ± SD. Cd: 5 μM, Fer-1: 15 μM, Erastin: 1 μM. ** *p* < 0.01, *** *p* < 0.001.

To explore ferroptosis’ function in Cd-induced cytotoxicity, we co-treated BEAS-2B cells with Fer-1 or Erastin and Cd for 24 h. The MTT assay findings demonstrated that Cd treatment markedly diminished cell viability compared to control group (*p* < 0.001). Notably, Fer-1 co-treatment attenuated the Cd-induced inhibition of cell viability, whereas Erastin co-treatment further exacerbated it (*p* < 0.001) (Figure 2A). The ROS test findings indicated a substantial elevation in intracellular ROS levels after cadmium exposure relative to the control group (*p* < 0.001). Fer-1 co-treatment effectively reduced ROS levels, while Erastin co-treatment led to a significant increase in ROS levels, in comparison with Cd treatment alone (*p* < 0.05) (Figure 2B,C). Additionally, the MMP detection results indicated that, in comparison to the control group, the Cd group reduced levels of MMP in BEAS-2B cells. Cd+Fer-1 treatment alleviated the decrease in MMP caused by Cd exposure, whereas Erastin co-treatment further diminished MMP, as compared with Cd treatment alone (*p* < 0.05) (Figure 3A,B). Moreover, iron ion tests revealed a significant increase in intracellular iron subjected to Cd exposure. While Fer-1 co-treatment reduced iron levels, Erastin co-treatment resulted in a further increase, in comparison with Cd treatment alone (*p* < 0.05) (Figure 3C). Western blot demonstrated that Cd exposure downregulated the levels of SLC7A11 and GPX4, crucial proteins associated in ferroptosis. Fer-1 co-treatment upregulated these proteins, whereas Erastin co-treatment downregulated them further, as compared with Cd treatment alone (*p* < 0.01) (Figure 4A–E). Collectively, the above findings highlight the critical significance of ferroptosis in Cd-induced BEAS-2B cell damage and suggest that modulating ferroptosis may influence Cd cytotoxicity.

### 2.4. The Nrf2/SLC7A11/GPX4 Pathway Is Implicated in Cd-Induced Ferroptosis in BEAS-2B Cells

**Figure 5:**
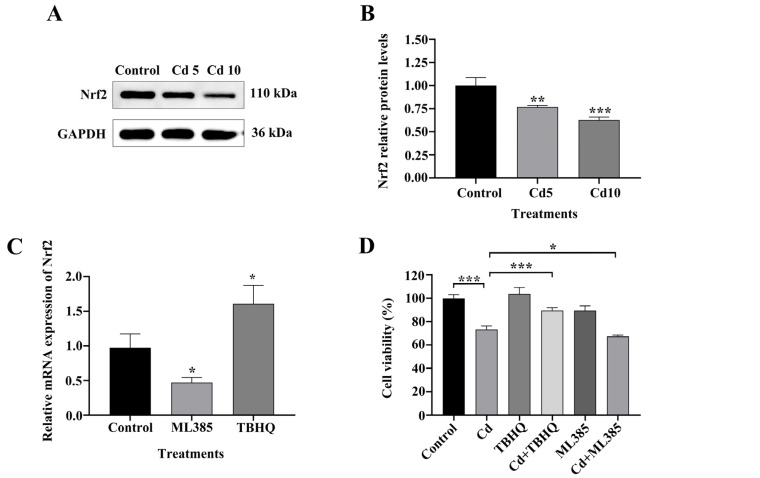

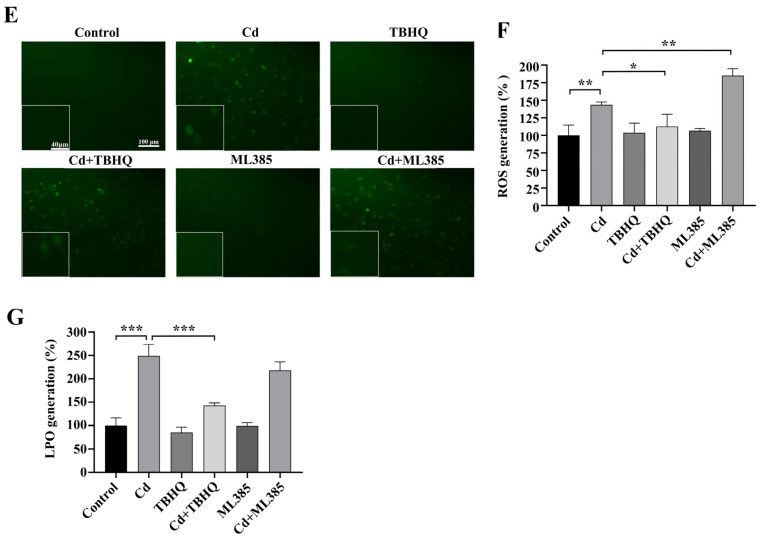
Regulation of Cd-Induced Ferroptosis in BEAS-2B Cells via the Nrf2/SLC7A11/GPX4 Pathway. (**A**,**B**) Protein expression of Nrf2 after 24 h of exposure to Cd. (**C**) RT-qPCR analysis of Nrf2 mRNA expression. (**D**) Cell viability assessed using MTT assay. (**E**,**F**) Intracellular ROS levels. (**G**) LPO content. The data is presented as the mean ± SD. Cd5: 5 μM Cd, Cd10: 10 μM Cd. TBHQ: 10 μM, ML385: 10 μM. * *p* < 0.05, ** *p* < 0.01, *** *p* < 0.001.

**Figure 6:**
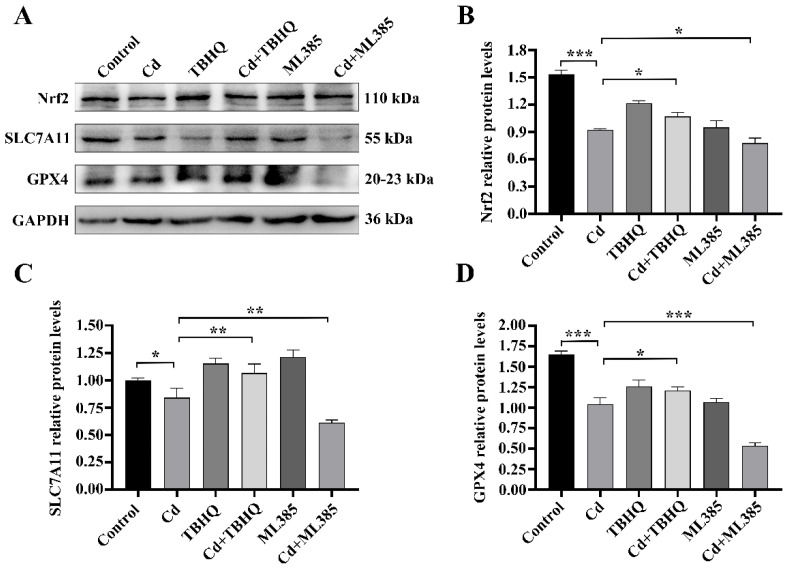
Effects of TBHQ and ML385 on Cd-Induced Modulation of the Nrf2/SLC7A11/GPX4 Pathway in BEAS-2B Cells. (**A**–**D**) Protein expression of Nrf2, SLC7A11, and GPX4 as determined by Western blot analysis. The data is presented as the mean ± SD. Cd: 5 μM, TBHQ: 10 μM, ML385: 10 μM. * *p* < 0.05, ** *p* < 0.01, *** *p* < 0.001.

We examined the involvement of ferroptosis in Cd-induced damage to BEAS-2B cells by evaluating Nrf2 expression and its downstream targets, SLC7A11 and GPX4, using western blot analysis. The findings indicated a significant decrease in Nrf2 protein levels after 24 h of Cd exposure compared to untreated controls (*p* < 0.01) (Figure 5A,B). Consistently, SLC7A11 and GPX4 expression also decreased with increasing Cd concentrations. Therefore, we hypothesized that Nrf2 depletion impairs the antioxidant capacity of BEAS-2B cells after Cd exposure, thereby promoting cell ferroptosis. To evaluate this hypothesis, we examined the function of Nrf2 in Cd-induced ferroptosis using BEAS-2B cells by regulating Nrf2 expression with the Nrf2 activator TBHQ and Nrf2 inhibitor ML385. To verify the sensitivity of BEAS-2B cells to TBHQ and ML385, we used RT-qPCR to detect changes in Nrf2 expression levels in cells treated with TBHQ or ML385 for 24 h. In comparison to the control group, TBHQ treatment markedly elevated expression levels of Nrf2, whereas ML385 significantly inhibited Nrf2 expression (*p* < 0.05) (Figure 5C). We then treated BEAS-2B cells with TBHQ or ML385 and Cd for 24 h and determined the corresponding indexes. The MTT assay results indicated that TBHQ co-treatment attenuated Cd-induced cytotoxicity and ML385 co-treatment exacerbated it (*p* < 0.05) (Figure 5D). We subsequently examined intracellular ROS and LPO levels to assess oxidative stress. The findings indicated that Cd exposure markedly elevated levels of ROS and LPO, TBHQ combined treatment decreased these levels, and ML385 combined treatment further increased ROS levels (*p* < 0.05) (Figure 5E–G). Furthermore, western blot analysis demonstrated that TBHQ co-treatment upregulated Nrf2, SLC7A11, and GPX4 expression; ML385 co-treatment had the opposite effect (*p* < 0.05) (Figure 6A–D). Collectively, these findings underscore the essential function of the Nrf2/SLC7A11/GPX4 pathway in Cd-induced ferroptosis in BEAS-2B cells. This indicates that altering this system may serve as a viable treatment approach for Cd-induced pulmonary injury.

### 2.5. Luteolin Attenuates Cd-Induced BEAS-2B Cell Injury by Inhibiting Ferroptosis

**Figure 7:**
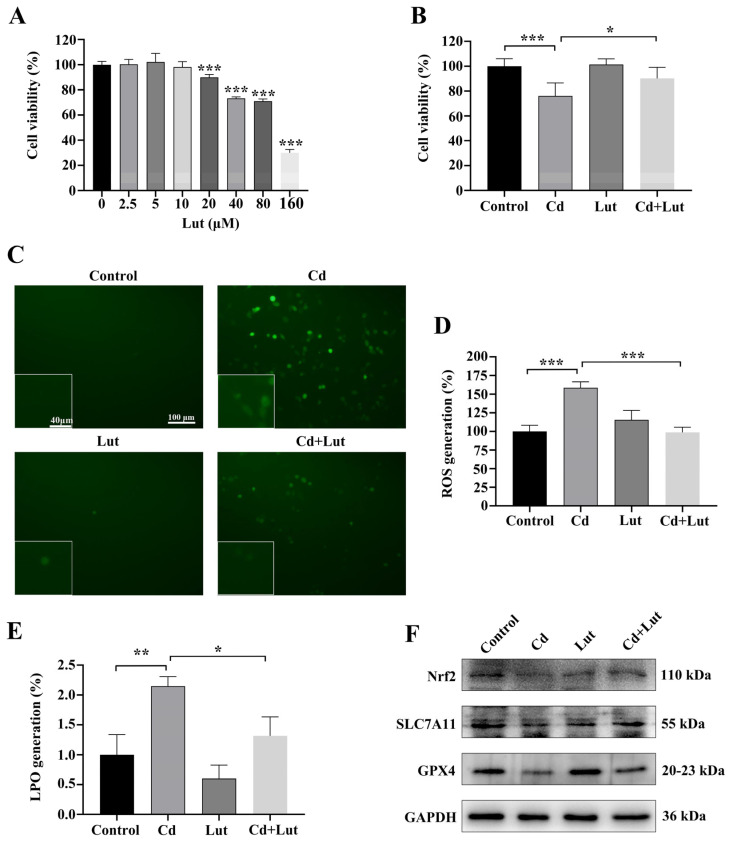

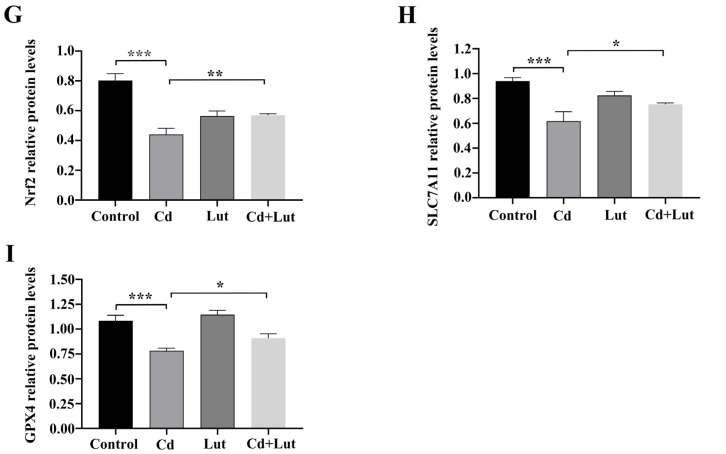
Luteolin Inhibits Cd-Induced Ferroptosis in BEAS-2B Cells. (**A**) After 24 h of treatment at different Lut concentrations (0, 2.5, 5, 10, 20, 40, 80, 160 μM), cell viability was evaluated by MTT test. (**B**) MTT experiment measuring cell viability after co-treatment with 5 μM Cd and 10 μM Lut for 24 h. (**C**,**D**) ROS generation was assessed using fluorescence microscopy. (**E**) LPO levels were measured after co-treatment with Cd and Lut. (**F**–**I**) Analysis of protein expression by western blot for Nrf2, SLC7A11, and GPX4. The data is presented as the mean ± SD. Cd: 5 μM, Lut: 10 μM. * *p* < 0.05, ** *p* < 0.01, *** *p* < 0.001.

We evaluated the cytotoxicity of Lut using the MTT test to ascertain the appropriate concentration for future investigations. The results indicated that 10 μM Lut did not impair viability in BEAS-2B cells (Figure 7A); this concentration was selected for further studies. To assess the protective impact of Lut against cytotoxicity triggered by Cd, cells received combined treatment with Cd and Lut for a period of 24 h. The MTT assay results demonstrated that Cd markedly reduced cell viability relative to the control group (*p* < 0.05). Co-treatment with Lut markedly attenuated Cd-induced loss of viability (*p* < 0.05) (Figure 7B). Intracellular ROS and LPO levels were measured to evaluate oxidative stress. Cd exposure significantly increased ROS and LPO levels, which were markedly reduced with Lut co-treatment (*p* < 0.05) (Figure 7C–E). Additionally, Western blot results showed Cd exposure reduced Nrf2, SLC7A11, and GPX4 levels. Lut co-treatment upregulated these proteins, indicating that the Nrf2/SLC7A11/GPX4 pathway was activated (*p* < 0.05) (Figure 7F–I). These findings suggested that Lut suppressed ferroptosis and stimulated the Nrf2/SLC7A11/GPX4 pathway to protect BEAS-2B cells from Cd-induced injury.

## 3. Discussion

Cd is a common environmental heavy metal that poses serious risks to the organs of humans and animals with chronic exposure [22,23]. Recent experimental studies identify ferroptosis—an oxidative cell death mechanism caused by iron dysregulation and lipid peroxidation—as a contributing component in neurological diseases (e.g., Alzheimer’s), ischemic tissue injury, and lung cancer development [24,25,26]. Our experimental findings indicated that Cd exposure triggers ferroptosis in BEAS-2B cells, as evidenced by multiple hallmarks: suppressed cell proliferation, excessive ROS generation, decreased MMP, accumulated LPO, and iron overload, coupled with diminished GSH-PX activity. Mechanistically, Cd-triggered ferroptosis is mediated through suppression of the Nrf2/SLC7A11/GPX4 antioxidant axis. Using an in vitro model of Cd injury, we identified treatment with Lut, a natural bioactive compound, as an effective intervention that can attenuate Cd-induced ferroptosis.

Experimental research indicates that ferroptosis is a crucial contributor to tissue damage induced by heavy metals [27,28,29]. Ferroptosis is directly linked to an imbalance in the intracellular iron metabolism. Under normal conditions, Fe^3+^ is transported into cells via transferrin and reduced to Fe^2+^ for use in physiological activities; excess Fe^2+^ is stored in ferritin to prevent its accumulation. Cells also maintain iron homeostasis by actively exporting Fe^2+^ via membrane iron transporters [30]. Exposure to the Cd can result in the abnormal accumulation of iron ions in cells by disrupting critical processes in iron metabolism, including the inhibition of ferritin’s storage function and the down-regulation of membrane iron transporters’ expression or activity [31]. The accumulation of iron can catalyze the production of significant quantities of ROS via mechanisms like the Fenton reaction, leading to lipid peroxidation, which is a critical factor in the initiation of ferroptosis [32]. In the present study, we measured intracellular iron levels in BEAS-2B cells following Cd exposure using an iron detection kit. The findings indicated a substantial elevation in intracellular iron levels after Cd exposure relative to the control group. The findings suggest that Cd exposure may interfere with iron homeostasis in BEAS-2B cells, resulting in abnormal iron accumulation. Since iron accumulation is a significant trigger of ferroptosis, it is hypothesized that ferroptosis could play a role in the injury process of BEAS-2B cells induced by cadmium.

Excessive ROS generation is strongly linked to ferroptosis [33,34]. Physiologically, ROS regulate cell proliferation, differentiation, migration, and programmed death [35]. However, excessive ROS can oxidize polyunsaturated fatty acids, generating LPO products [36]. This study revealed that Cd exposure markedly elevated ROS and LPO levels in BEAS-2B cells. GSH-PX is an antioxidant enzyme that inhibits LPO using GSH to convert lipid peroxides into non-toxic lipid alcohols. Our results showed that Cd markedly decreased the activity of GSH-PX in BEAS-2B cells relative to the control group. This result suggested that by inhibiting GSH-PX activity, Cd exposure weakens the antioxidant defense of cells, leading to an increase in levels of ROS and LPO. Excess ROS also impair mitochondrial structure and function, reducing MMP and consequently inducing ferroptosis [37,38]. In this study, Cd exposure significantly reduced MMP in BEAS-2B cells, indicative of mitochondrial dysfunction. Collectively, the above findings suggested that Cd upregulates ferroptosis in BEAS-2B cells by promoting ROS generation and inducing mitochondrial damage.

To confirm ferroptosis’s role in Cd-triggered BEAS-2B cell toxicity, we modulated ferroptosis using the specific inhibitor Fer-1 and the activator Erastin. Our findings showed that Cd-exposed BEAS-2B cells co-treated with Fer-1 had significantly higher viability than those treated with Cd alone. Conversely, cells co-treated with Cd and Erastin showed a significant decrease in viability. Fer-1 significantly attenuated the Cd-induced increases in ROS and iron ion levels and ameliorated mitochondrial damage; in contrast, Erastin exacerbated these changes. Collectively, these findings further confirm that Cd induces BEAS-2B cell injury via the activation of ferroptosis.

GPX4 serves as a marker protein for ferroptosis, catalyzing the reduction of phospholipid hydroperoxides to phospholipids; its inactivation results in the buildup of phospholipid hydroperoxides, thereby initiating ferroptosis [39]. SLC7A11 is a key subunit of the System Xc^−^ transporter that maintains GSH biosynthesis by mediating cystine/glutamate antiport, thereby facilitating GPX4-dependent lipid peroxide clearance [40]. In this study, Cd exposure markedly reduced SLC7A11 and GPX4 protein expression in BEAS-2B cells. Moreover, intervention with Fer-1 or Erastin revealed that Fer-1 attenuated the Cd-induced downregulation of SLC7A11 and GPX4, whereas Erastin exacerbated it. These findings indicated that Cd induces ferroptosis by suppressing the function of the System Xc^−^ transporter, thereby reducing cellular cystine uptake and diminishing GPX4-mediated lipid peroxide detoxification, which disrupts the cellular antioxidant defense mechanism.

Nrf2 is a master transcriptional regulator of antioxidant defense that regulates ferroptosis and maintains redox homeostasis [41]. When Nrf2 function is inhibited, ferroptosis-related proteins SLC7A11 and GPX4 exhibit downregulated expression [42]. Feng et al. demonstrated that triptolide induces cervical cancer cell ferroptosis and inhibits tumor growth by reducing Nrf2 expression, which in turn suppresses GPX4 and SLC7A11 expression and increases LPO [43]. Consistent with this finding, we revealed that Cd exposure significantly downregulated Nrf2 protein expression in BEAS-2B cells. We hypothesize that Nrf2 may regulate ferroptosis by modulating SLC7A11 and GPX4 expression. To test this hypothesis, we used the Nrf2 activator TBHQ and the inhibitor ML385 to modulate Nrf2 expression and investigate its role in Cd-induced ferroptosis. Our results showed that Cd exposure markedly downregulated Nrf2, SLC7A11, and GPX4 expression. TBHQ co-treatment upregulated these proteins, whereas ML385 co-treatment further downregulated them. These results confirmed that the Nrf2 signaling pathway is critical in suppressing Cd-induced ferroptosis in BEAS-2B cells and enhancing cellular antioxidant capacity. Thus, bioactive compounds that specifically activate the Nrf2/SLC7A11/GPX4 pathway and have good safety profiles may offer a novel therapeutic strategy for Cd-associated pulmonary toxicity.

**Figure 8:**
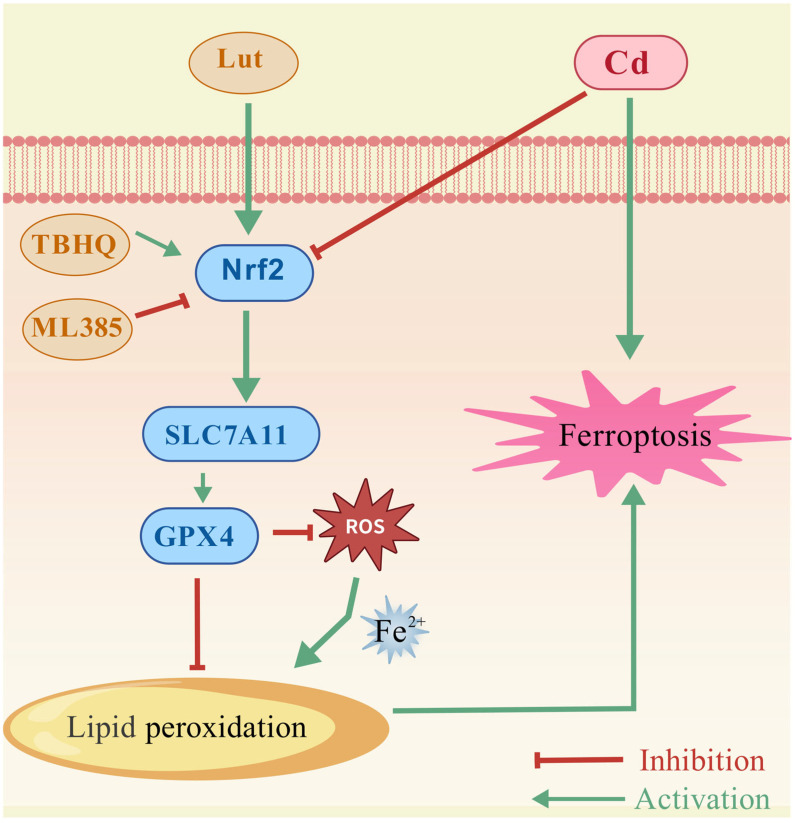
Potential mechanism of Cd-induced ferroptosis in Beas-2B cells.

Natural bioactive compounds, particularly flavonoids, are widely recognized for their preventive effects against disease [44,45,46,47], exhibiting great potential in alleviating heavy metal toxicity [48]. Lut is a common flavonoid found in many plants, and it possesses multiple beneficial activities [49]. Notably, Lut has been shown to mitigate the combined toxicity of various heavy metals in HL7702 liver cells by inhibiting ROS-mediated mitochondrial apoptosis pathways, thereby reducing cell death [50]. Additionally, Lut can inhibit ferroptosis by targeting the NR4AL/SLC7A11/GPX4 pathway, thereby alleviating renal injury and fibrosis induced by calcium oxalate crystals [51]. Wang et al. demonstrated that Lut, along with baicalin, can block key ferroptosis pathways by inhibiting ROS accumulation, upregulating GPX4 expression, and downregulating Acsl4/Ptgs2 gene expression, thereby alleviating myocardial ischemia/reperfusion injury [52]. GPX4 is a significant downstream target gene of the transcription factor Nrf2, and Nrf2 activation serves as a fundamental defense mechanism for cells against oxidative stress and ferroptosis [53]. Recent studies suggest that flavonoids can activate Nrf2 via various mechanisms, including the direct modification of a specific cysteine residue on its inhibitory protein Keap1. This modification induces conformational changes in Keap1, resulting in the release of Nrf2, which subsequently translocates into the nucleus to initiate transcription [54]. Additionally, flavonoids may enhance the stability and activation of Nrf2 by modulating the activities of upstream kinase pathways, including MAPK and PI3K/Akt [55,56]. Moreover, considering that Nrf2 is highly responsive to REDOX states, the strong antioxidant properties of Lut significantly lower the ROS levels caused by Cd and may also indirectly enhance the stability and activation of Nrf2 by mitigating oxidative stress. However, It is still uncertain if Lut can inhibit ferroptosis triggered by environmental heavy metal Cd, particularly regarding its potential involvement in the regulation of the Nrf2 pathway. Our study findings showed that Lut attenuated Cd-induced suppression of BEAS-2B cells viability and significantly reduced intracellular ROS and LPO levels following Cd exposure. Additionally, Lut increased Nrf2 expression along with that of its downstream proteins SLC7A11 and GPX4. Based on the results presented, it is proposed that Lut may effectively inhibit ferroptosis in BEAS-2B cells induced by Cd exposure through the activation of the Nrf2/SLC7A11/GPX4 signaling pathway, thereby enhancing cellular antioxidant defense and inhibiting lipid peroxidation (Figure 8).

This study systematically elucidates, for the first time, the molecular mechanism by which Cd exposure induces ferroptosis through the inhibition of the Nrf2/SLC7A11/GPX4 pathway, resulting in lung epithelial cell damage and offering a theoretical foundation for investigating lung protection strategies centred on pathway modulation. This research has certain limitations: Initially, the deleterious effects of Cd exposure on pulmonary tissue and associated processes were not substantiated in animal models; Secondly, the in vivo protective efficacy of Lut has not been assessed in animal pharmacodynamic studies. Future research will include in vivo investigations, including the creation of a Cd-exposed mice model, and will further validate the findings of this study using lung tissue pathological examination, molecular marker assessment, and Lut intervention experiments.

## 4. Materials and Methods

### 4.1. Cell Culture

Human bronchial epithelial cells (BEAS-2B, ATCC) were cultivated in DMEM medium (Gibco, Grand Island, NY, USA) augmented with 5% fetal bovine serum (FBS). Cells were cultured at 37 °C in a humidified environment with 5% CO_2_. Upon achieving 70–80% confluence, the cells were treated as follows: (1) Control (cells only); (2) 1.25, 2.5, 5, 10, 20, 40 μM CdCl_2_ for 24 h; (3) 15 μM Fer-1 or 1 μM Erastin for 24 h; (4) CdCl_2_+ Fer-1 co-treatment (5 μM CdCl_2_ + 15 μM Fer-1, 24 h); (5) CdCl_2_ + Erastin co-treatment (5 μM CdCl_2_ + 1 μM Erastin, 24 h); (6) 10 μM TBHQ for 24 h; (7) 10 μM ML385 for 24 h; (8) CdCl_2_ + TBHQ co-treatment (5 μM CdCl_2_ + 10 μM TBHQ, 24 h); (9) CdCl_2_ + ML385 co-treatment (5 μM CdCl_2_ + 10 μM ML385, 24 h); (10) 10 μM Lut for 24 h; (11) CdCl_2_+Lut co-treatment (5 μM CdCl_2_ + 10 μM Lut, 24 h). Cells were harvested for downstream analyses.

### 4.2. Reagents and Antibodies

CdCl_2_ was sourced from Sigma-Aldrich (St. Louis, MO, USA). Lut came from Yuanye Biotechnology (Shanghai, China). Iron detection and LPO/GSH-PX assay kits were acquired from Applygen Technology (Beijing, China) and Nanjing Jiancheng Bioengineering Institute (Nanjing, China), respectively. The JC-1 mitochondrial detection kit is from Beyotime Biotechnology (Shanghai, China). TRIzol reagent was obtained from Ambion (Austin, TX, USA). Ferrostatin-1, Erastin, and ML385 were purchased from MedChemExpress (Monmouth Junction, NJ, USA), while TBHQ was from Yuanye Biotechnology (Shanghai, China). Antibodies targeting Nrf2, GPX4, SLC7A11, and GAPDH, along with IP/Western Lysis Buffer, were supplied by Proteintech Group (Wuhan, China).

### 4.3. MTT Assay

BEAS-2B cells were seeded at 4 × 10^3^/well, treated with various CdCl_2_ doses (0, 1.25, 2.5, 5, 10, 20, 40 μM) for 24 h, then incubated with MTT for 4 h. Supernatant was removed, and 100 μL DMSO solubilized formazan crystals, which were measured by microplate readers (Thermo Fisher Scientific, St. Louis, MO, USA) at 490 nm [57].

### 4.4. ROS Detection

BEAS-2B cells in the logarithmic growth phase were chosen and inoculated onto 6-well plates at a density of 1 × 10^6^/mL for overnight cultivation. Subsequent to cell adhesion, they were subjected to several doses of CdCl_2_ (0, 5, 10 µM) independently for 24 h, or to CdCl_2_ (5 µM) in conjunction with the intervention reagent for 24 h. Eliminate the original cell culture media, do a single wash with PBS, introduce 1 mL of diluted DCFH-DA to each well (1 μL of DCFH-DA: 1000 μL of serum-free culture medium), and thereafter incubate in a 37 °C cell incubator for 30 min. The cells were rinsed thrice with serum-free cell culture media and thereafter examined under a fluorescence microscope. The fluorescence intensity of the cells was assessed via Image J software [58].

### 4.5. Detection of Iron Ions

BEAS-2B cells in the logarithmic growth stage were inoculated at an appropriate density in 10 cm culture dishes and cultured overnight. They were treated alone with different concentrations of CdCl_2_ (0, 5, 10 µM) for 24 h or co-treated with CdCl_2_ (5 µM) and Fer-1 (or Erastin) for 24 h. After discarding the original DMEM cell culture medium, the cells were washed twice with pre-cooled PBS. Add 100–200 μL of lysis buffer to each dish. Use a cell scraper to rapidly transfer the cells in the culture dish into the labeled EP tubes, shake vigorously for 20–30 s, place on a shaker for lysis for 2 h, and then centrifuge at 12,000× *g* rpm for 5 min using a low-temperature high-speed centrifuge pre-cooled at 4 °C. After centrifugation, mix the supernatant with an equal volume of iron buffer and incubate at 60 °C for 1 h. Then, at room temperature, add 30 μL of iron detection reagent and incubate for 30 min. Then, the detection was carried out using an microplate reader at 550 nm.

### 4.6. Intracellular LPO Detection

BEAS-2B cells in the logarithmic growth phase were injected onto 10 cm culture plates at suitable quantities and incubated overnight. The subjects were administered several dosages of CdCl_2_ (0, 5, 10 µM) for 24 h or treated with CdCl_2_ (5 µM) with the intervention reagent for 24 h. Subsequent to rinsing the cells twice with pre-cooled PBS, introduce 100–200 µL of lysis buffer to each plate. Utilise a cell scraper to efficiently move the cells from the culture plate into designated EP tubes. Incubate the EP tubes on ice for 30 min to facilitate cell lysis. Subsequent to lysis, position the EP tubes in a low-temperature, high-speed centrifuge at 4 °C. Centrifuge at 12,000× *g* revolutions per minute for 30 min. Subsequent to centrifugation, put the supernatant into a 1.5 mL EP tube. Prepare empty tubes, standard tubes, and test tubes in accordance with the instructions. Upon completion of the preparation, immerse them in a 45 °C water bath for 60 min. Centrifuge at 4000× *g* rpm for 10 min after the water bath. Transfer 200 μL of the supernatant to a 96-well plate. The absorbance readings for each well were measured at a wavelength of 586 nm using a microplate reader.

### 4.7. Intracellular GSH-PX Detection

BEAS-2B cells in the logarithmic growth phase were injected at a suitable density in 10 cm culture plates and incubated overnight. They were treated alone with different concentrations of CdCl_2_ (0, 5, 10 µM) for 24 h or treated with CdCl_2_ (5 µM) in combination with the intervention reagent for 24 h. Following two washes of the cells with pre-cooled PBS, introduce 100–200 µL of lysis buffer to each plate. Utilise a cell scraper to efficiently move the cells from the culture plate into designated EP tubes. Incubate the EP tubes on ice for 30 min to facilitate cell lysis. After lysis, place the EP tubes in a low-temperature high-speed centrifuge at 4 °C Centrifuge at 12,000× *g* rpm/min for 30 min. Following centrifugation, extract the supernatant and transfer it to a new EP tube, then perform the enzymatic reaction according to the specified instructions. Upon completion of the enzymatic reaction, the EP tube is centrifuged at 4000× *g* rpm for 10 min, and 1 mL of the supernatant is extracted for the colour development reaction. Upon completion of the colour development reaction, thoroughly mix the EP tubes and allow them to stand at ambient temperature for 15 min. Transfer 200 μL of the supernatant to a 96-well plate. The absorbance value of each well at a wavelength of 412 nm was measured using an microplate reader.

### 4.8. MMP Assay

BEAS-2B cells in the logarithmic growth phase were selected and inoculated into 6-well plates at a density of 1 × 10^6^/mL for overnight culture. After the cells adhered, they were treated with different concentrations of CdCl_2_ (0, 5, 10 µM) alone for 24 h or with CdCl_2_ (5 µM) in combination with the intervention reagent for 24 h. After the culture is completed, take out the 6-well plate from the cell incubator, discard the original culture medium, and wash it once with PBS buffer (1 mL per well). Subsequently, add 1 mL of cell culture medium and 1 mL of JC-1 staining working solution (prepared in the ratio of 5 μL of JC-1 to 1 mL of staining buffer) to each well, mix thoroughly, and then place the 6-well plate back in the cell incubator and incubate for 20 min. After incubation, discard the supernatant, wash twice with staining buffer, then add 2 mL of cell culture medium to each well and observe under an inverted fluorescence microscope.

### 4.9. Real-Time Quantitative Polymerase Chain Reaction (RT-qPCR)

Total RNA was extracted from cells with TRIzol. DNA was synthesized with a reverse transcription kit and fluorescent RT-qPCR with UltraSYBY Mixture. Table 1 lists the PCR primer sequences.

### 4.10. Western blot

The IP/Western Lysis Buffer, which includes phosphatase and protease inhibitors, lysed the cells. Protein concentrations were quantified with the BCA Assay. Following the equalisation of protein amounts using SDS-PAGE, we transferred them on PVDF membranes. We subsequently permitted primary antibodies specific to Nrf2 (1:1000), GPX4 (1:1000), SLC7A11 (1:1000), and GAPDH (1:50,000) to incubate overnight at 4 °C on membranes pre-blocked with 5% non-fat milk. Then, the PVDF membranes washed by TBST were transferred to HRP-labeled sheep anti-rabbit IgG and sheep anti-mouse IgG secondary antibodies (1:12,000), and incubated at room temperature for 1 h. Ultimately, we examined the protein bands with ImageJ 8.0 software and an ECL apparatus.

### 4.11. Statistical Analysis

The statistics are expressed as mean ± standard deviation from three independent experiments. The statistical study did one-way analysis of variance. The test level was α = 0.05, and *p* < 0.05 was considered to indicate statistical significance.

## 5. Conclusions

In summary, This work comprehensively established, for the first time in an in vitro cell model, the molecular mechanism by which Cd exposure induces ferroptosis via the inhibition of the Nrf2/SLC7A11/GPX4 pathway, resulting in lung epithelial cell destruction. In addition, this study also found that the natural flavonoid compound Lut can effectively antagonize Cd-induced cell ferroptosis by activating the Nrf2/SLC7A11/GPX4 pathway, offering novel intervention targets and prospective tactics for the prevention and treatment of Cd-related lung toxicity.

## Figures and Tables

**Table 1:** Primer sequences.

Name	Forward Primer	Reverse Primer
*GAPDH*	CGCTCTCTGCTCCTCCTGTT	CCATGGTGTCTGAGCGATGT
*NRF2*	AGGACATGGAGCAAGTTTGG	TCTGTCAGTGTGGCTTCTGG

## Data Availability

The data presented in this study are available on request from the authors.

## Abbreviation

**Full Name**	**Abbreviation**
American Type Culture Collection	ATCC
Cadmium	Cd
Dimethyl sulfoxide	DMSO
Ferrostatin-1	Fer-1
Glutathione Peroxidase 4	GPX4
Reduced glutathione	GSH
Glutathione Peroxidase	GSH-PX
Lipid peroxidation	LPO
Luteolin	Lut
Mitochondrial membrane potential	MMP
Methyl Thiazolyl Tetrazoliun	MTT
Nuclear factor erythroid-2 related factor 2	Nrf2
Phosphate buffered saline	PBS
Reactive oxygen	ROS
Sodium dodecyl sulphate	SDS
Solute carrier family 7 member 11	SLC7A11
Tert-Butylhydroquinone	TBHQ
Tris(hydroxymethyl)aminomethane	Tris
